# Supporting Migrant 2SLGBTQIA+ Unpaid Caregivers for Family Members Living with Chronic Illnesses

**DOI:** 10.3390/healthcare13131533

**Published:** 2025-06-27

**Authors:** Roya Haghiri-Vijeh, Robin Coatsworth-Puspoky, Harish Ramesh, Arvin Shakibai, Willian Roger Dullius, Marcus Allan

**Affiliations:** 1 School of Nursing, Faculty of Health, York University, Toronto, ON M3J 1P3, Canada; 2 Department of Chemistry, Faculty of Science, York University, Toronto, ON M3J 1P3, Canada; 3 School of Kinesiology & Health Sciences, Faculty of Health, York University, Toronto, ON M3J 1P3, Canada; 4 Serviço de Enfermagem de Psiquiatria Infantil e de Adição (SEPIA), Hospital de Clínicas de Porto Alegre (HCPA), Porto Alegre 90035-903, RS, Brazil; 5 School of Health, Nursing Course, ATITUS Educação, Porto Alegre 90035-903, RS, Brazil; 6 Waypoint Center for Mental Health Care, Penetanguishene, ON L9M 1G3, Canada; 7 Department of Psychiatry, Division of Geriatric Psychiatry, University of Toronto, Toronto, ON M5T 1R8, Canada

**Keywords:** caregivers, 2SLGBTQIA+, migrants, older adults, chronic illness

## Abstract

The literature details the healthcare needs of migrant people living with chronic illnesses and the consequent economic, social, and healthcare needs of their caregivers. Similarly, some studies have underscored the social and healthcare needs of 2SLGBTQIA+ (two-spirit, lesbian, gay, bisexual, transgender, queer, and intersex individuals, including diverse sexual and gender identities under the “+” symbol) adults living with chronic illnesses and their caregivers. This narrative review presents the context of migrant 2SLGBTQIA+ unpaid caregivers and how their intersecting identities influence their caregiving roles for family members with chronic illnesses. In this article, caregivers are defined as family members or chosen families who provide unpaid support that may last for three months or longer for people living with chronic illnesses. Most studies and policies overlook 2SLGBTQIA+ migrants who are also unpaid caregivers of individuals living with chronic illnesses, leaving them unsupported through discrimination at the intersection of racism, homophobia, transphobia, ageism, and ableism, forcing them to remain vulnerable to increased emotional and physical strain. There is a presence of pervasive systemic barriers, including a lack of training and education among social and healthcare providers, about the needs of migrant 2SLGBTQIA+ unpaid caregivers. Additional challenges stem from inadequate policies and insufficient targeted resources, particularly for caregivers from marginalized racial and ethnic backgrounds. The findings of this study highlight the necessity for a call to action to address these gaps and improve support systems for these highly marginalized communities.

## 1. Introduction

Collins [1] and Crenshaw [2] define intersectionality as when intersecting identities of an individual, such as race, gender identity and expression, sexual orientation, sex characteristics, socioeconomic status, [dis]ability, and age result in compounded forms of discrimination. People who identify as two-spirit, lesbian, gay, bisexual, transgender, queer, and diverse sexual orientations, gender identities and expressions, and sex characteristics (2SLGBTQIA+) often experience discrimination and stigma in having their health and social care needs met [3]. To add another layer of complexity, 2SLGBTQIA+ migrants have experienced non-affirming social and healthcare services where their needs for their intersecting identities were not met [4]. Yet, 2SLGBTQIA+ migrants may take on the role of unpaid caregivers for friends, family, and chosen family. For migrant 2SLGBTQIA+ unpaid caregivers who provide physical, emotional, and/or financial support to a friend, family, or chosen family in need, these intersecting forms of discrimination and non-affirming care shape their healthcare and caregiving experiences. Marginalization of caregivers’ identities are further compounded by systemic barriers within the healthcare system through policies that fall short of addressing the specific needs of the caregiver and the person they are caring for [5,6,7].

Healthcare providers, such as nurses, doctors, and special therapists, are trained professionals who offer various medical, therapeutic, and/or supportive health and social services for clients with chronic illnesses; however, they may not have been educated about the intersecting needs of equity-deserving communities. Insufficient training among healthcare providers on the specific needs of migrant 2SLGBTQIA+ unpaid caregivers and how to address them can result in a lack of knowledge, contributing to increased stress for these caregivers [6,8,9]. Furthermore, many organizational policies have led to direct and indirect discrimination against 2SLGBTQIA+ unpaid caregivers [10], resulting in their inability to provide care for their family members. Direct discrimination against 2SLGBTQIA+ unpaid caregivers can include organization policies that explicitly prevent them from making medical decisions for their partners or chosen family members. Indirect discrimination can occur when policies fail to recognize chosen family structures, making it more difficult for unpaid caregivers to advocate for their loved ones. An unpaid caregiver’s ethnic background can contribute to discrimination when cultural biases or systemic barriers result in unequal access to healthcare resources, language barriers, or assumptions about family caregiving roles. Discrimination against 2SLGBTQIA+ caregivers can also take the form of ageism, where younger caregivers may be dismissed or not taken seriously, or ableism, where caregivers with disabilities face additional challenges in providing care [8]. Ageism and ableism can lead to physical and emotional distress, and an increased burden on caregivers, ultimately affecting both their well-being and the quality of care they can provide. Despite facing compounded discrimination, migrant 2SLGBTQIA+ unpaid caregivers provide care and manage their responsibilities. Addressing systemic barriers would further support their well-being and financial security, allowing them to continue their vital caregiving roles with greater ease [8,11,12].

There is an urgent need for systemic changes, such as culturally sensitive training for healthcare providers and tailored resources, to meet the needs of 2SLGBTQIA+ migrant unpaid caregivers [11]. These positive changes can help alleviate inequities and foster a more inclusive and affirming caregiving environment. In this article, we focus on migrant 2SLGBTQIA+ individuals who are unpaid caregivers of their family members living with chronic illnesses. We begin by drawing on the concept of intersectionality to frame this article. We then provide background information on 2SLGBTQIA+ migrants as unpaid caregivers and explore the development of care circles, including family care dynamics. The discussion also addresses the need for training healthcare professionals, the role of support groups, and the importance of collecting SOGIESC-related data on this topic. SOGIESC stands for sexual orientation, gender identity and expression, and sex characteristics. Finally, we outline directions for future research and discuss policy implications.

## 2. Methods

To explore the challenges faced by 2SLGBTQIA+ migrant caregivers, a narrative review was conducted. This type of review allows for an in-depth description and interpretation of previously published literature on a selected topic. One of the advantages of conducting a narrative review is its flexible yet rigorous approach to analyzing and interpreting the literature [13]. Although this is a usable and relevant approach to the inquiry, this is not a systematic review and lacks a protocol; therefore, we critically acknowledge the potential for selection bias in this review.

This narrative review was conducted to explore the unique challenges faced by migrant 2SLGBTQIA+ unpaid caregivers supporting family members with chronic illnesses. Given the complexity of intersecting identities and the systemic barriers that these caregivers face, a narrative review allows for identifying broader themes and patterns across multiple sources. It also provides insight into existing knowledge gaps and informs future research directions. A thorough but focused examination of existing literature was conducted while accounting for recent developments in healthcare policies, migration patterns, and intersectionality frameworks. Members of the team gathered information based on the barriers to and enablers of 2SLGBTQIA+ unpaid caregivers of family members with all types of chronic diseases. The sources used explored the intersectionality of 2SLGBTQIA+ caregivers and the challenges they face while providing care for family members with chronic illnesses.

We included peer-reviewed journal articles, policy reports, systematic reviews, and fact sheets from organizations specializing in 2SLGBTQIA+ caregiving, chronic illness care, and intersectionality. In February 2025, after consulting a health science librarian and utilizing York University’s library search engine, we searched for sources with any publication date to the present. A variation of the following keywords was employed: 2SLGBTQIA+, LGBT*, migra*, immigra*, newcomer, care provider*, family*, health*. Some additional key search terms included 2SLGBTQIA+ caregivers, 2SLGBTQIA+ caregivers of Alzheimer’s disease family members, issues faced by 2SLGBTQIA+ caregivers, 2SLGBTQIA+ caregivers of chronically ill family members, and inequalities among 2SLGBTQIA+ caregivers. No specific search period was defined, given the thematic relevance and the scarcity of publications on the subject. These sources include empirical studies on the challenges faced by 2SLGBTQIA+ caregivers, e.g., [8,14], and advocacy-based reports addressing systemic healthcare inequities, e.g., [8,14]. Furthermore, theoretical works on intersectionality, e.g., [1,15,16], and aging-related caregiving experiences, e.g., [11] were included to provide an extensive understanding of the topic. Additionally, details were recorded about unique caregiving concerns, such as avoiding potential bias in healthcare settings and stigmatizing those in caregiving roles.

## 3. Intersectionality and Minority Stress Model as a Grounding Framework

The term “intersectionality” was used in Black feminist literature, predating Kimberlé Crenshaw’s more widely recognized use of the term to highlight inequalities affecting Black women due to the intersection of sexism and racism [17]. In 1989, Kimberlé Crenshaw highlighted how oppressed social groups live on the margins of society, experiencing unequal access to resources and opportunities. Crenshaw explored how various social identities intersect and interact to shape individuals’ experiences and opportunities. The scholars using intersectionality as a framework to emphasize that systems of oppression, such as racism, sexism, classism, ableism, and 2SLGBTQIA+-phobia, are interconnected and cannot be understood in isolation [4,15,18]. They argue that these individual identities overlap, intersect, and reflect macro-level forms of oppression and privilege [1,16].

The intersectionality theory is summarized in three principles: (i) social identities are not independent but multiple and interconnecting; (ii) individuals from historically oppressed and marginalized groups are the focal point; and (iii) intersectionality can help reveal disparate health outcomes [19]. Addressing human rights violations against 2SLGBTQIA+ people through an intersectional perspective will help move toward meaningful and comprehensive advances. It demonstrates the various ways that intersectionality can be used and how it has the potential to reveal interlocking relationships of oppression and privilege, exposing how experiences of 2SLGBTQIA+ people vary due to the intersections of gender, ethnicity, class, religion, birthplace, politics, and other societal contexts [20].

The Minority Stress Model describes the collective experiences of individuals with diverse gender and sexual identities and orientations, including those shaped by intersectional differences. This model is conceptualized through distinct yet interconnected components of stress: distal stress (external experiences such as discrimination and aggression) and proximal stress (internal experiences such as self-critical beliefs). Both types of stress can negatively affect mental health and interpersonal functioning [4]. However, the impact of 2SLGBTQIA+ minority stress may be mediated by individual resilience and the presence of social support [21]. The intersectionality of multiple identities held by unpaid caregivers can also cause compounding stress within their lives.

## 4. 2SLGBTQIA+ Migrants as Unpaid Caregivers

Unpaid caregivers are defined as individuals such as family members, friends, or other support persons (i.e., neighbors) who provide health and/or social care services for an individual, but they are not compensated for their time [14,22,23]. Unpaid caregivers provide support to meet care recipients’ care needs because of their “personal relationship” with them [24,25]. The care needs of recipients include aspects related to aging and physical, intellectual, developmental, medical and mental health conditions [25,26]. Often unpaid caregivers have no formal training or support in providing these unpaid services [23] as it is not a job [26], in contrast to paid or formal healthcare providers [24]. Often, unpaid caregivers are in female gender roles [14,24], reinforcing the “cultural model” of caregiving. The cultural model identifies caregiving as part of the unpaid caregivers’ commitment or obligation to care for the care recipient, reinforces the inaccuracies of the “non-essential” nature of the old term of “informal caregiving,” and may hinder unpaid caregivers from seeking knowledge about their caregiving activities [24]. As a result, unpaid caregivers may not self-identify as a caregiver or may not identify their need for support or inclusion in healthcare activities or decision-making [24]. In some cases, unpaid caregivers may also be HCPs, but they are providing this support for their family members without any pay and only in their capacity as family members [14].

Discrimination against 2SLGBTQIA+ unpaid caregivers can occur across various societal systems, including healthcare settings, workplaces, and broader social structures, due to factors such as age, gender, ethnicity and/or race. Migrant 2SLGBTQIA+ unpaid caregivers of color may have to navigate issues within the cultures of their country of origin or face practices that are inherently discriminatory toward their intersectional identity. Society’s phobias toward these caregivers can lead to more health disparities between the caregiver and the care recipient [14]. Many healthcare professionals assume that caregivers are cisgender or heterosexual [7,22]. Cisgender refers to one’s gender identity and expression aligning with their reported sex at birth [25], whereas heterosexuality refers to being attracted to the opposite sex. Heterosexual men would be attracted to heterosexual women and vice versa [26]. The assumption of cisgenderism or heterosexuality by healthcare providers leads to an indirect dismissive atmosphere for 2SLGBTQIA+ caregivers [22] and creates spaces where unpaid caregivers feel excluded and unsafe [6,7].

Many migrant 2SLGBTQIA+ unpaid caregivers may not have been given legal recognition, especially when caring for non-biological family members or partners. Without formal legal documentation, migrant 2SLGBTQIA+ unpaid caregivers might lack authority in making medical and financial decisions for their loved ones [27]. This lack of recognition can lead to the exclusion of unpaid caregivers from critical decisions [14,27]. This may include being questioned about whether they are unpaid caregivers or could carry the responsibility of being unpaid caregivers [23]. Not only healthcare providers but also biological family members may disown or not recognize the 2SLGBTQIA+ person as a valid member of their family. This lack of acceptance may lead some biological families to exclude or take responsibility away from the migrant 2SLGBTQIA+ unpaid caregiver who is providing care for a chronically ill family member [14]. This can be in the form of taking away their decision-making capabilities as unpaid caregivers or compounding more discrimination within the family dynamic [22]. The overarching stress of having their responsibility as unpaid caregivers being undermined in many sections of society can cause anxiety and an emotional toll [28]. Hence, there are legal challenges regarding caregiving responsibilities. Families may also discriminate against the caregiver and may legally remove decision-making from the migrant 2SLGBTQIA+ unpaid caregivers.

Many unpaid caregivers who are either Black or part of the 2SLGBTQIA+ community, and who provide care for adults with Alzheimer’s disease, report experiencing financial difficulties [29]. Black 2SLGBTQIA+ caregivers are under-represented, underserved, and they face many financial strains and societal barriers [29]. These economic barriers within this caregiver community can manifest in the form of struggling to purchase medications for their family members. This constant compounding fear of not being able to afford necessary medications can cause high levels of stress [14,29]. Migrant 2SLGBTQIA+ unpaid caregivers of chronically ill relatives face compounded discrimination across legal, familial, healthcare, and financial systems, where their caregiving roles are often overlooked or undermined due to intersecting identities, leading to increased emotional stress, economic hardship, and health disparities for the caregiver [14,22]. Addressing the unique needs of migrant 2SLGBTQIA+ unpaid caregivers, especially those with experiences of migration, requires a comprehensive approach that acknowledges both the diversity within this community and the structural challenges they face. This comprehensive approach must consider the social, legal, and systemic barriers these unpaid caregivers encounter, as well as the ageism and ableism prevalent in healthcare systems and 2SLGBTQIA+ spaces, which further compound the challenges of navigating the support and resources required.

## 5. Care Circles

### 5.1. Chosen Family

One of the primary ways to address the needs of migrant 2SLGBTQIA+ unpaid caregivers is to provide migration and SOGEISC-affirming spaces that acknowledge the chosen family in the circle of care [11]. Many 2SLGBTQIA+ individuals rely on a network of support that extends beyond traditional family structures, often referred to as “chosen family” [8,30]. Embracing this broader care circle with an equity lens is crucial, as it allows each member of the caregiving team to be treated with the respect and authority they need to offer effective support. Recognizing the legitimacy of these care circles in legal and medical contexts can alleviate many of the obstacles 2SLGBTQIA+ unpaid caregivers face, including exclusion from critical caregiving decisions [31]. Migrants often form chosen families in their resettlement countries. These chosen families can help them navigate the challenges of resettlement and foster a sense of belonging in new environments [31,32]. The chosen families provide emotional support and practical assistance, helping individuals adapt and thrive [33]. Moreover, 2SLGBTQIA+ refugees have chosen families that help them feel understood, safe, and welcomed into a new country [34]. Individuals connected through a chosen family also feel safer and have a stronger social bond. Hence, it would be beneficial to recognize and prioritize migrant 2SLGBTQIA+ unpaid caregivers’ chosen families [35]. Having discussed the crucial role of chosen family, we suggest that healthcare professionals incorporate a caregiver burden assessment for 2SLGBTQIA+ migrants who have taken on this unpaid role. Several caregiver burden assessment tools, such as the Zarit Burden Interview, the Caregiver Burden Inventory, the Caregiver Burden scale, the Relatives Stress Scale [36,37], and the Caregiver Strain Index [38], are available. Morgan et al. [39] examined the differences in caregiving strain levels between older person caregivers who were sexual and gender minorities (SGM) and those who were cisgendered using the caregiver strain index [38]. Researchers found that caregivers who were gay or lesbian (SGM status) was not only predictive of caregiver strain, but it was greater than the strain experienced by cisgendered or heterosexual caregivers. According to findings from a scoping review completed by Hall et al. [6], 2SLGBTQIA+ unpaid caregivers experience high levels of stress (emotional, physical, psychological, social, and financial), which places them at high risk for becoming overwhelmed in their caregiving role. Stressful conditions may affect the unpaid caregiver and, in turn, impact the quality of care they provide. To have a more comprehensive understanding of both the caregiver and care recipient, tools such as the Subjective Burden Scale may be helpful [37] and demonstrate the need to support 2SLGBTQIA+ unpaid caregivers [6]. Yet, due to limited integration of these tools with 2SLGBTQIA+ migrants, we encourage healthcare clinicians and scientists to employ an evidence-informed approach [40] and consider further evaluation of the relevance of these tools for these unpaid caregivers.

### 5.2. Health Care Provider Training

An intersectional approach is equally important in providing holistic support to 2SLGBTQIA+ caregivers. Scholars, utilizing intersectionality as a lens, have emphasized the social and healthcare needs of diverse communities and acknowledged the compounded challenges that caregivers experience when underserved individuals belong to multiple marginalized groups [41]. For instance, 2SLGBTQIA+ unpaid caregivers who are also racial minorities [29,35] or migrants [41] often face layers of discrimination, from cultural stigma to limited access to resources, due to systemic biases in healthcare and social services. Research shows that the emotional state of caregivers has a direct influence on the quality of care provided to the care receiver [42]. Valenti and Katz [42] highlight that increased stress, invisibility, and lack of recognition can exacerbate emotional distress in caregivers. This often leads to caregiver burnout. This experience of burnout may reduce the attentiveness and quality of care available to the care recipient. Incorporating a critical ethnography and intersectionality research and teaching approach would allow us to better understand how unpaid caregivers impact the person receiving care. To address these compounded issues, we suggest increased training for healthcare professionals on the nuances of intersectionality in caregiving [42].

We acknowledge that education alone is not sufficient, but it is the first step in informing healthcare professionals’ understanding of the experiences and needs of migrant 2SLGBTQIA+ unpaid caregivers. First, educators in healthcare professional programs need to ensure they are well-versed in this content and not project their biases and assumptions onto their students. For example, in a recent study, we found that a registered nurse educator for personal support workers in Canada used 2SLGBTQIA+-phobic language while discussing culturally safe care [4]. This is concerning as a majority of care in long-term care facilities for older adults is delivered by personal support workers. In this study, a nonbinary migrant nurse who had experienced judgment and discrimination from nurses both as a patient and nursing colleagues shared their worry that they will be one of those older adults who will end up having to go back into the closet. Some of the participants were not sure if non-migrant 2SLGBTQIA+ healthcare providers would understand their migration trajectory, 2SLGBTQIA+ experiences, language barrier, and experiences of racism and ageism. In ref. 43 they were also afraid of how they would be perceived by non-2SLGBTQIA+ care providers, residents, and visitors within long-term care facilities [44]. Therefore, it is important that educators of interdisciplinary healthcare professionals also undergo positive space training to ensure they are equipped to provide unbiased education to future healthcare professionals [3].

Second, the intersecting needs of migrant 2SLGBTQIA+ unpaid caregivers need to be included in the education of healthcare professionals. By centering trauma-and-violence-informed practices, educators should draw on unique narratives and living experiences of migrant 2SLGBTQIA+ unpaid caregivers for current and future healthcare providers. The minority stress model helps healthcare professionals not only accept migrant 2SLGBTQIA+ unpaid caregivers but also strive for opportunities to improve the support migrant 2SLGBTQIA+ caregivers require [7,44,45]. Integration of narratives and living experiences of migrant 2SLGBTQIA+ migrants can lead to a more empathetic response by healthcare providers when working within these communities [45]. Stinchcombe et al. [7] assert that 2SLGBTQIA+ unpaid caregivers caring for persons with dementia need safe environments that are free of stigma and discrimination. Having their experiences and identities affirmed, recognized as inconsistent with heteronormative aging expectations, and understood and shared by others were necessary to reduce feelings of exclusion from dementia care [6]. To mobilize the voices and experiences of 2SLGBTQIA+ older persons and aging in gerontological education, Lipinski et al. [9] constructed digital stories of three 2SLGBTQIA+ community members. In two of the three stories, older persons identified concerns about aging and caregiving in their relationship. In educational sessions, participants identified that the stories raised their awareness, knowledge, and understanding about 2SLGBTQIA+ older persons’ experiences of aging. Participants also expressed their desire to support changes in policies and practices for 2SLGBTQIA+ older persons. Embedding information about the challenges of migrant 2SLGBTQIA+ unpaid caregivers in healthcare provider curricula can make these unpaid caregivers feel more welcome in a healthcare environment [44,45].

While improving training for healthcare providers on intersectionality in caregiving is an important first step, education alone cannot ensure competent and inclusive care [4]. Structural changes that improve support systems for migrant 2SLGBTQIA+ unpaid caregivers should accompany awareness efforts to create a welcoming environment for migrant 2SLGBTQIA+ caregivers. For example, healthcare organizations, governing bodies, accrediting bodies, practice colleges and associations may consult experts on migrant 2SLGBTQIA+ issues to help revise policies and curricula to ensure they are inclusive and supportive of migrant 2SLGBTQIA+ unpaid caregivers. This can help build a strong foundation for future improvements that move beyond cultural competence and into cultural safety and humility practices.

### 5.3. Support Groups

Migrant 2SLGBTQIA+ unpaid caregivers navigate the intersectional challenges from both their migrant and 2SLGBTQIA+ identities and orientations. In 2SLGBTQIA+ communities, establishing support groups specifically tailored for these intersectional identities can offer a safe space for migrant 2SLGBTQIA+ unpaid caregivers to feel recognized and valued and help to mitigate the emotional toll associated with their caregiving role [46]. For migrant caregiving communities, it has been found that support groups have significantly lowered stress and decreased feelings of isolation and in turn act as a protective factor [45]. Moreover, support groups for migrant caregivers looking after family members with psychotic disorders have been shown to be beneficial to the well-being of the caregiver. 2SLGBTQIA+ caregivers have also reported improved livelihoods and lower stress once integrated into a support group [47]. In a Canadian study, it was found that 2SLGBTQIA+ migrants asked for support groups that were specific for their intersecting identities of 2SLGBTQIA+ and migration status versus general support groups that did not address their intersecting identities [4]. In particular, some of the participants in this study highlighted that their experiences were diverse and not all of the 2SLGBTQIA+ migrants should be assimilated into the same support group. For example, support groups need to be tailored for the experience of 2SLGBTQIA+ non-documented refugees versus 2SLGBTQIA+ international students [4]. Given the important and positive impact of support groups on migrant caregivers, 2SLGBTQIA+ migrants, and 2SLGBTQIA+ caregivers, it stands to reason that migrant 2SLGBTQIA+ unpaid caregivers would similarly benefit from these tailored spaces. Therefore, specific support groups can be beneficial for this diverse, unpaid caregiver population.

### 5.4. SOGIESC Data Collection

To enhance the quality and specificity of care, a model of inclusive data collection related to sexual orientation, gender identity and expression, and sex characteristics (SOGIESC) [3] is recommended for migrant 2SLGBTQIA+ individuals in unpaid caregiving roles. SOGIESC data refers to self-reported information about an individual’s sexual orientation, gender identity, gender expression, and sex characteristics, providing a more comprehensive understanding of individuals’ identities and orientations [48]. SOGIESC data can be collected through electronic means such as short surveys. It is important to note that SOGIESC data is not constant for an individual; therefore, collecting SOGIESC data at multiple points in time can be useful. The ease of access to electronic surveys can help supplement the multiple collections of SOGIESC data [49]. To better understand and address the unique challenges faced by migrant 2SLGBTQIA+ unpaid caregivers, it is essential to recognize how SOGIESC data intersects with their experiences. Migrant communities, often navigating multiple layers of marginalization, can benefit from more nuanced data collection that reflects their diverse identities and orientations. However, there is a lack of comprehensive data on the SOGIESC identities of these individuals. Without accurate data, there is a risk that resources may not be appropriately allocated to support these unpaid caregivers, leaving gaps in care and exacerbating existing disparities. By doing so, healthcare providers can gain insights into the particular needs and challenges faced by migrant 2SLGBTQIA+ unpaid caregivers, allowing for more targeted and affirming support. Sharing this data with healthcare providers can reduce the burden on caregivers by helping to tailor care plans to better meet the needs of both unpaid caregivers and care recipients [29,50,51,52]. Additionally, combating ageism and ableism within 2SLGBTQIA+ spaces, as well as tackling homophobia and transphobia in spaces for adults, will further address the often-overlooked needs of aging 2SLGBTQIA+ caregivers and their care recipients [6]. For migrant 2SLGBTQIA+ unpaid caregivers, these structural and social barriers are further compounded by challenges related to migration status, language accessibility, and cultural stigma surrounding both migrant and 2SLGBTQIA+ identities. Without inclusive data collection and targeted policies, their experiences are rendered invisible, leading to inadequate support. Prioritizing their inclusion in data-driven decision-making is a necessary step toward ensuring that healthcare and social systems recognize and address their unique needs [40].

## 6. Future Research

Due to limited research on the needs of migrant 2SLGBTQIA+ unpaid caregivers, future research should prioritize the development of specific resources for migrant 2SLGBTQIA+ unpaid caregivers. For example, researchers may focus on how best to offer gender-affirming, accessible, and inclusive support for migrant 2SLGBTQIA+ unpaid caregivers. This future research could take a community-based participatory research approach to involve migrant 2SLGBTQIA+ unpaid caregivers in shaping the research priorities, causing the research to be more relevant to the specific caregiver population [40]. In addition, a longitudinal study regarding the needs of migrant 2SLGBTQIA+ unpaid caregivers can be beneficial, allowing the response to policy changes or support systems to be tracked over time [40]. Community-based participatory research and other approaches to future research can produce data to help inform future policies to meet the needs of migrant 2SLGBTQIA+ unpaid caregivers and how best to support them. Future research may also involve creating an advisory board to review caregiver stress, burden, and strain measures with older persons who identify as 2SLGBTQIA+ to determine and validate whether the measured items fit with the unpaid caregivers’ burden, stress, and strain. Alternatively, research may include exploring 2SLGBTQIA+ older persons’ burden/stress/strain and adapting a measure to fit caregivers’ needs.

## 7. Implications for Policy Makers

Mobilizing knowledge about the distinct experiences, resilience, strengths, and challenges of migrant 2SLGBTQIA+ unpaid caregivers, especially those navigating intersectional identities, can help to inform policy changes and foster a more inclusive approach within healthcare professions and unpaid caregivers. These policy changes can occur at the level of healthcare and social services to ensure culturally welcoming spaces for intersectional caregivers [3,6]. An example of a potential policy change would be to gather admission information about the chosen family in the medical decision-making process as opposed to only the biological family [30]. Additionally, lobbying for funding agencies to support research and initiatives centered on migrant 2SLGBTQIA+ unpaid caregivers is crucial. Ensuring these caregivers are actively included in the decision-making process and policy development can lead to more equitable support systems.

## 8. Conclusions

Unpaid 2SLGBTQIA+ caregiving experiences were influenced by caregiver psychosocial factors such as emotional well-being, social support, and support from the 2SLGBTQIA+ communities [6,22,39,53,54,55]. Caregivers’ emotional well-being was influenced by support from families of choice and friendships, and the members of the 2SLGBTQIA+ communities were critical for reducing caregiver stress and maintaining their health [6,22]. This support assisted caregivers in coping with families of origin and healthcare providers’ failure to recognize or accept them as the caregiver, and also with violence (misgendering and mistreatment) related to older persons’ gender identities [6,22,53]. Caregivers’ feelings of fear, guilt, regret, unsafety, and the need to make changes for the recipient’s care resulted from experiencing and witnessing cisgenderist violence, such as disrespect, degradation, reduction to “transness,” being a “freak show,” mistreatment, and denial of gender affirming treatments, against care recipients [53]. Caregivers were “counted on” to support the care recipients’ gender expression outwardly in clothing, hairstyle, or binding and inwardly in access to and administration of medications. Similarly, Morgan et al. [39] found that care recipients’ sexual identity increased or decreased caregiver strain. However, when recipients were part of the SOGIESC minority communities, caregivers’ strain was reduced by 2.5 points on the caregiver scale (aIRR + 0.67: CI 0.55, 0.80). This reduction in caregiver strain suggests that SOGIESC minority communities’ support is unique and decreases caregiver emotional stress and accumulated SOGIESC minority caregiver strain. This strain may include victimization, poor physical health, poor relationships and social networks, and limited access to health care [55], p. 758; strain and support that respite services [55] and bereavement support groups [54] do not understand and therefore are unable to address for 2SLGBTQIA+ caregivers. To recognize the need for inclusivity, support of SOGIESC identities and disclosure, and promote the health and well-being of 2SLGBTQIA+ caregivers and recipients, 2SLGBTQIA+ caregivers constructed their own community support networks [22].

Intersectionality presents compounded challenges for migrant 2SLGBTQIA+ unpaid caregivers, which may hinder their ability to provide effective care for their chronically ill family members. Discrimination in family or healthcare settings based on the unpaid caregiver’s SOGIESC can lead to increased stress within the caregiver and mental health challenges. This group has faced a variety of stigma and discrimination in their lives, which are amplified by their contextual positionalities [4,7]. Moreover, there is a lack of policies and support systems for migrant 2SLGBTQIA+ unpaid caregivers, leading to economic constraints, which add to their already stressful lives [5]. This article contributes to the existing body of research by focusing on the unique challenges faced by migrant 2SLGBTQIA+ unpaid caregivers and the need to support them. Previous studies have explored challenges faced by 2SLGBTQIA+ caregivers or migrant caregivers separately. Yet, this article highlights the compounded barriers that arise at the intersection of the 2SLGBTQIA+ identity and the migrant identity. Additionally, we shift the focus onto the unpaid caregivers themselves, rather than the care recipient, underscoring unpaid caregivers’ specific needs and the systemic changes necessary to support them.

On the societal level, the next steps to tackle challenges faced by migrant 2SLGBTQIA+ unpaid caregivers can include increasing the visibility of their experiences and needs. Focusing on the societal level is vital as community-based initiatives may have a direct and meaningful impact on the daily lives of these unpaid caregivers. This can help amplify the representation of the migrant 2SLGBTQIA+ unpaid caregivers, allowing more opportunities to challenge discrimination and decrease systemic inequalities [7]. More community-based research is needed to construct specific and helpful resources with and for 2SLGBTQIA+ unpaid caregivers that are focused on intersectionality and are congruent with their migrant status, thus helping to support their unpaid caregiving and reducing any excessive economic or mental burden.

## Data Availability

No new data were created in this study.

## Abbreviations

The following abbreviations are used in this manuscript:

2SLGBTQIA+	Two-Spirit, lesbian, gay, bisexual, transgender, queer, intersex, asexual, and the + stands for identities and orientations not named in the acronym, yet equally important.
UCs	Unpaid caregivers
SOGIESC	Sexual orientation, gender identity and expression, and sex characteristics.
